# Perioperative Risk Stratification: A Need for an Improved Assessment in Surgery and Anesthesia—A Pilot Study

**DOI:** 10.3390/medicina57101132

**Published:** 2021-10-19

**Authors:** Bianca-Liana Grigorescu, Irina Săplăcan, Marius Petrișor, Ioana Roxana Bordea, Raluca Fodor, Alexandra Lazăr

**Affiliations:** 1 Department of Pathophysiology, University of Medicine, Pharmacology, Sciences and Technology, 540142 Târgu-Mureș, Romania; bianca.grigorescu@umfst.ro; 2 Department of Anesthesiology and Intensive Care, Emergency County Hospital, 540136 Târgu-Mureș, Romania; 3 Department of Simulation Applied in Medicine, University of Medicine, Pharmacology, Sciences and Technology, 540142 Târgu-Mureș, Romania; marius.petrisor@umfst.ro; 4 Department of Oral Rehabilitation, University of Medicine and Pharmacy Iuliu Hațieganu, 400012 Cluj-Napoca, Romania; 5 Department of Anesthesiology and Intensive Care, University of Medicine, Pharmacology, Sciences and Technology, 540142 Târgu-Mureș, Romania; raluca.fodor@umfst.ro (R.F.); alexandra.lazar@umfst.ro (A.L.)

**Keywords:** Acute Physiology and Chronic Health Evaluation (APACHE II), Physiological and Operative Severity Score for the enumeration of Mortality and morbidity (P-POSSUM), Surgical APGAR Score (SAS), morbidity, mortality, perioperative risk assessment, surgery, anesthesiology

## Abstract

*Background and Objectives*: Numerous scoring systems have been introduced into modern medicine. None of the scoring systems assessed both anesthetic and surgical risk of the patient, predict the morbidity, mortality, or the need for postoperative intensive care unit admission. The aim of this study was to compare the anesthetic and surgical scores currently used, for a better evaluation of perioperative risks, morbidity, and mortality. *Material and Methods*: This is a pilot, prospective, observational study. We enrolled 50 patients scheduled for elective surgery. Anesthetic and surgery risk was assessed using American Society of Anesthesiologists (ASA) scale, Physiological and Operative Severity Score for the enumeration of Mortality and morbidity (P-POSSUM), Acute Physiology and Chronic Health Evaluation (APACHE II), and Surgical APGAR Score (SAS) scores. The real and the estimated length of stay (LOS) were registered. *Results*: We obtained several statistically significant positive correlations: ASA score–P-POSSUM (*p* < 0.01, r = 0.465); ASA score–SAS, (*p* < 0.01, r = −0.446); ASA score–APACHE II, (*p* < 0.01 r = 0.519); predicted LOS and ASA score (*p* < 0.01, r = 0.676); predicted LOS and *p*-POSSUM (*p* < 0.01, r = 0.433); and predicted LOS and APACHE II (*p* < 0.01, r = 0.454). A significant negative correlation between predicted LOS, real LOS, ASA class, and SAS (*p* < 0.05) was observed. We found a statistically significant difference between the predicted and actual LOS (*p* < 001). *Conclusions*: Anesthetic, surgical, and severity scores, used together, provide clearer information about mortality, morbidity, and LOS. ASA scale, associated with surgical scores and severity scores, presents a better image of the patient’s progress in the perioperative period. In our study, APACHE II is the best predictor of mortality, followed by P-POSSUM and SAS. P-POSSUM score and ASA scale may be complementary in terms of preoperative physiological factors, providing valuable information for postoperative outcomes.

## 1. Introduction

The performance and quality of care provided to patients could be assessed in terms of recovery and mortality regarding a specific pathology [1]. Accurate clinical judgment, the risk of adverse outcomes derived from available data and severity of illness should be quantified in a scoring system that helps physicians to assess the efficiency of the treatment provided to their patients [2,3]. The first assessment of medical treatment outcomes was performed in 1863 by Florence Nightingale, and it was based on the personal judgment of the clinician [4,5]. Rapid improvement of medical and surgical procedures as well as the development of intensive care units (ICU) enhanced the need for quantitative and clinically relevant outcome measures that could assess the effectiveness of a treatment [6]. In the last few decades, many scoring systems with high screening performance were introduced in modern medicine. They were tailored to predict patient outcome, to evaluate the efficiency of treatments and to convert the severity of an illness in a quantifiable variable [7,8]. Despite this situation, even though there are a lot of scoring systems for ICU, surgery, or emergency medicine, none of them comprise all the variables involved in perioperative period assessment of patients who undergo anesthesia and surgery [9,10].

None of the scoring systems developed along the time assess both anesthetic and surgical risk of the patient, as well as predicting the morbidity, mortality, and the need for postoperative ICU admission. Four systems for calculating postoperative mortality have incorporated the American Society of Anesthesiologists (ASA) physical status: The Surgical Risk Scale, The American College of Surgeons National Surgical Quality Improvement Program (ACS NSQIP), The Surgical Outcome Risk Tool, and The National Emergency Laparotomy Audit [11].

The ASA classification system was first introduced in 1941 and it represents a method used to assess patient operative risk on a scale from 1 to 5 (6 being attributed to brain dead patients). Because it is based on variables that quantify the physical status of the patient, it can be used as an effective risk stratification metric to predict medical complications, morbidity, and mortality in postoperative period [12]. ASA score is a measure of patient’s well-being. Patients with higher ASA classes are at risk of developing higher rates of postoperative medical complications, requiring advanced therapeutic measures or ICU admission, and associated higher mortality [13,14].

Portsmouth - Physiological and Operative Severity Score for the enumeration of Mortality and morbidity (P-POSSUM) was proposed as a good predictor of post-operative outcome in surgical procedures. This score is a reliable tool for determining the quality of care given to a patient to avoid post-operative complications that might lead to increased morbidity and mortality [15,16].

Surgical APGAR Score (SAS) is a 10-point scale that predicts postoperative outcomes and is also considered a potential tool for the prediction of postoperative ICU admission [17,18]. It was first proposed it in 2007 as a straightforward method of evaluating patients after general or vascular procedures based on three intraoperative variables: lowest heart rate, lowest mean arterial pressure, and anticipated blood loss. The SAS has yet to be shown in the field of liver transplantation. One reason could be because in liver transplantation, the estimated blood loss (EBL) category typically meets or exceeds the SAS maximum score (1000 mL) [19].

Because of its simplicity and capacity to categorize severity of disease and predict hospital mortality, the Acute Physiology and Chronic Health Evaluation (APACHE II) analysis has been applied in many ICUs across the world since 1985. Although it is not a surgical or anesthetic score, it is one of the most comprehensive and can provide a complex and accurate picture of a patient’s clinical and biological status, allowing for mortality prediction [20,21].

The aim of this pilot study was to compare the most used scoring systems in current anesthetic and surgical practice for a better evaluation of intra- and postoperative risks, as well as morbidity and mortality, expressed as the need for ICU admission and hospital length of stay (LOS).

## 2. Materials and Methods

Our study is a pilot, prospective, observational, and ongoing study, conducted in the Emergency Clinical County Hospital of Târgu Mureș. We enrolled 50 consecutive patients scheduled for elective surgery, aged between 23 and 81 years. This study is conducted with the approval of the ethics commission: number 18681/23.07.2021. The GDPR agreement was respected, and the data obtained were used only for research purposes.

Inclusion criteria: patients aged above 18 years; scheduled for elective surgery (general surgery, orthopedics, gynecology); without neurological and psychiatric disorders.

Exclusion criteria: patients under 18 years; scheduled for cardio-vascular, obstetrics, and neurosurgery; emergency surgery; recent previous multiple hospital admissions; previous prolonged ICU length of stay, surgical reinterventions, andneurological or psychiatric disorders.

For each patient, we gathered demographic information, time of surgery, and laboratory test results. Anesthetic risk was assessed using ASA, P-POSSUM, APACHE II Surgical SAS, scores were calculated prior surgery for each patient. For every enrolled patient the LOS, in accordance with the scoring scales, was calculated. Both–the real and the estimated–LOS were registered. Data were collected in a database and were analyzed using SPSS Statistics v. 17 for Windows. Alpha value for statistical significance threshold was set to 0.05. Data series normality was tested using Kolmogorov–Smirnov test.

Correlations between scores values were analyzed using Spearman correlation test. Mortality rates were compared using Friedman test.

## 3. Results

Our study involved 24 males and 26 women; the average age was 57 ± 15 years, time of surgery ranges between 25–370 min, median was 95 min (IQR: 90).

We obtained the following median values for the studied surgical and severity scores: P-POSSUM: 19 (IQR: 12.25), APACHE II: 8.5 (IQR: 6), SAS: 8 (IQR: 1). Median for ASA score was 3 (IQR: 1).

**Figure 1:**
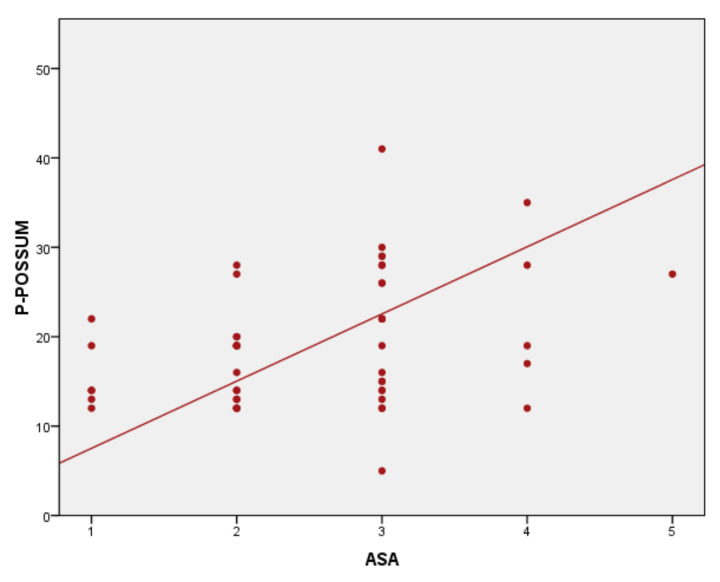
American Society of Anesthesiologists (ASA) score–Physiological and Operative Severity Score for the enumeration of Mortality and morbidity (P-POSSUM) score correlation.

**Figure 2:**
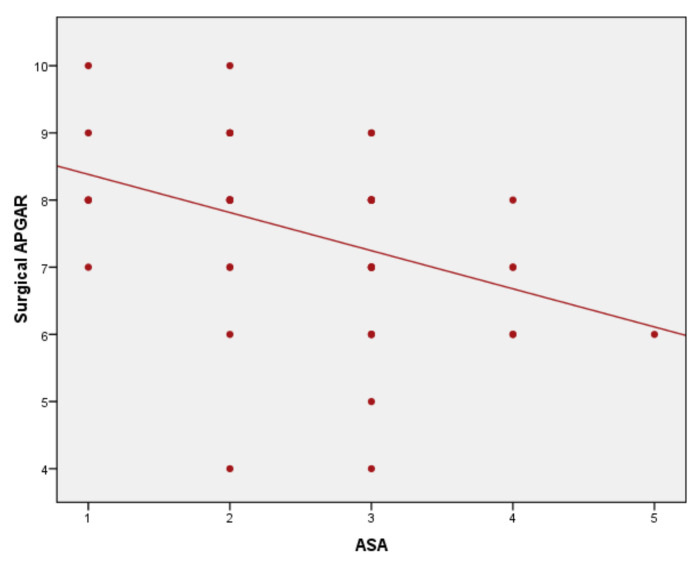
ASA score–Surgical APGAR Score (SAS) correlation.

**Figure 3:**
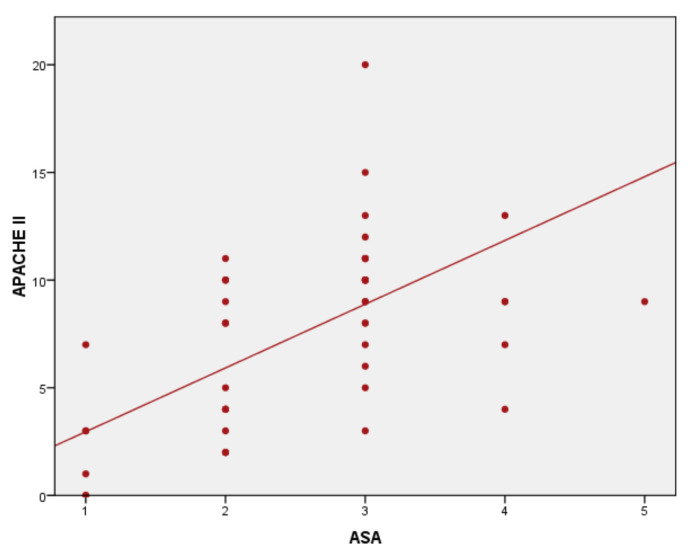
ASA score–Acute Physiology and Chronic Health Evaluation (APACHE II) correlation.

Results of correlation analysis of ASA score to severity and surgical scores are statistically significant, with *p* < 0.01 and correlation coefficients varying from 0.45 to 0.51. The ASA score–P-POSSUM correlation was statistically significant (*p* < 0.001), with a correlation coefficient r = 0.465 (Figure 1). The ASA score–SAS correlation was statistically significant, *p* < 0.001 and the correlation coefficient r = −0.446 (Figure 2), as well as correlation ASA score–APACHE II with *p* < 0.001 and r = 0.519 (Figure 3).

We found a statistically significant difference between the predicted and actual LOS, median values were 4 days for predicted LOS and 9 days for real LOS, *p* < 0.001 (Table 1). We also found a significant correlation between predicted LOS, surgical scores and ASA score, *p* < 0.001. All three deceased patients were diagnosed with metastasis neoplastic disease and the surgical interventions were scheduled and performed with palliative purpose. For the deceased patients ASA risk was 3–5, APACHE II varied between 9 and 30 points (9–30% mortality), P-POSSUM 17-20 (2.3–22% mortality), and SAS 6-8 points (1–4% mortality).

The analysis for the estimated mortality of P-POSSUM, APACHE II, and SAS is displayed in Table 2. Friedman testing, used to compare these variables, showed a statistically significant difference between them (*p* < 0.01).

The statistical analysis showed a significant positive correlation between predicted LOS and POSSUM score, APACHE II score, and ASA class (*p* < 0.01) (Table 3).

A statistically significant negative correlation between predicted LOS, real LOS, ASA class, and SAS (*p* < 0.05) was found (Table 4).

## 4. Discussion

Nowadays, to improve the quality of medical care for patients undergoing surgery, it is mandatory to use an accurate scoring system that comprises all the variables involved in the complex anesthetic and surgical procedures.

A scoring system normally has two components: a score—a number attributed to the severity of the condition; and a probability model—an equation giving the probability of hospital death of the patients [5]. Despite their different grades of complexity, all scoring systems used in anesthesia and surgery are prone to errors. This could be explained by the involved variables that quantified the clinical status of the patients. These findings are more evident in ASA scoring, because it does not require high laboratory tests. This is consistent to Alex Helkin et al. study that suggested that there is a risk of misclassification of the ASA score. In their study, in cases of elective surgery, the ASA score was underestimated; while in emergency surgery, the trend was to overestimate the ASA score. It is unknown why ASA ratings were incorrectly labeled. They believe that it was attributable to the fact that the study was developed in a teaching hospital, and younger anesthesia trainees are not as familiar with the ASA score calculation as they should be, or the emergency cases were handled at night and on weekends [22]. De Cassai et al. suggest in their study that the more experienced anesthesiologists recorded the lowest number of correct answers. The highest number of correct answers was obtained by residents [23]. To exclude possible errors in assigning the ASA class in our study, the ASA score was performed by the senior anesthetist with the utmost care. The ASA score has been explicitly validated for use in the NSQIP being an important predictor of death in surgical patients [24]. ASA physical status evaluation is imperfect; however, when integrated with additional clinical variables in systems like the Surgical Outcome Risk Tool (SORT), National Emergency Laparotomy Audit (NELA), ACS NSQIP scores, and frailty scores, it can assist accurately in patient outcome prediction [11].

According to the NSQIP online preoperative risk calculator, a misclassified ASA score significantly changed predicted mortality [25].

There are several factors during the operative period that can influence the evolution of the patient, which cannot be predicted by the ASA score. Potential perioperative complications could be related to blood loss, hypotension, and bradycardia. Surgery related risk factors include extent of incision, emergent indication, and wound class [26].

ASA weakness to predict perioperative risk on its own, highlights the need for other severity scores to have a broader view of perioperative complications, length of stay (LOS) in the ICU, morbidity, and risk of death [27]. Despite its importance in assessing postoperative morbidity and mortality, the ASA score cannot evaluate some particularities of the trans-surgery period that could jeopardize the life of patients and request high qualified medical care in ICU [28]. This is the reason why several surgical and severity scoring systems were developed; however, despite their complexity, none of them comprise all the risk factors involved in a complex anesthetic-surgical procedure. In this study, we obtained positive correlations between the ASA scale and the surgical scores. Although used together, they can provide us with a broader view of the condition of the patient and possible complications, this is difficult to apply in daily practice. As a result, to address both surgical and anesthetic needs, higher comprehensive scores are required [11]. For a better assessment of postoperative outcomes in surgical patients we used both anesthetic and surgical scoring systems. A peculiarity of our study is related to the correlation between APACHE II score (used as a predictor of mortality for ICU patients) and the aforementioned scoring system.

P-POSSUM score can be used by surgical teams to analyze and enhance the quality of surgical care, resulting in better patient outcome by avoiding predicted difficulties in surgical cases [2,29]. Its strength consists of its structure which is divided in two parts: the preoperative physiological factors and the surgical data collected during the trans- and postoperative periods. The physiological component of the score consists of 12 variables separated into four levels with exponential scores of 1, 2, 4, and 8. The variables consist of signs and clinical symptoms of each patient, results of biochemical examinations, hematologic investigation, and electrocardiographic examinations, which can provide a more extensive and reliable picture of the perioperative period [29].

We found a positive correlation between P-POSSUM score and ASA scale, thus the aforementioned scores might be complementary in terms of preoperative physiological factors, providing valuable information for postoperative outcomes.

Hopkins et al., in their study based on a cohort of 700,000 patients, state that ASA class correlates with the risk of 48-h mortality following an anesthetic procedure [30]. In our study the results showed a negative correlation between SAS and ASA scale because a high SAS score means a better prognosis, while a high ASA score means a patient with more comorbidities. In terms of its simplicity, the SAS score could be considered as the ASA score, allowing us to opinionate that it is a surgical variant of the ASA score used in the postoperative period [14,31]. In an attempt to create a new scoring system, that would enable a comprehensive assessment of preoperative and intraoperative patient status, M. Kinoshita et al. proposed in their study the SASA score. SASA is a combination of ASA score and SAS. In comparison to the ASA score and SAS, the SASA score offers superior predictability in terms of 30-day mortality [32]. According to several studies, the POSSUM scoring system is a stronger predictor of mortality than the APACHE II scoring system [33]. In our study, in terms of mortality, APACHE II is the best predictor of mortality, followed by P-POSSUM and SAS. This could be explained by the fact that both P-POSSUM and APACHE II assess physiological status and presence of organ dysfunctions, providing a more accurate physical status assessment of the patients and can predict their potential to develop postoperative complications [34]. This is in concordance with a study published by Kisa N.G. et al. which found that in patients undergoing oncological gastrointestinal surgery the APACHE II score and the P-POSSUM score are more reliable than the ASA scoring system in predicting mortality [35].

The APACHE II score was initially tailored to predict the mortality risk for intensive care patients. This score, which ranges from 0 to 71 points, is a typical instrument for assessing the severity in ICUs [36]. To offer a broad disease severity assessment, the APACHE II uses a point score based on initial values of 12 regular physiological parameters, patient age, and medical history. The APACHE II scoring system is ideal for intensive care patients but requires 24 h of observation and weighting tables for individual illness states [7,37]. Despite its initial destination, we used this scoring system in our study since several intraoperative events could decompensate or aggravate a previous medical condition. In these situations, a ‘surgical’ patient becomes an intensive care one, and a rigorous assessment of his condition is mandatory.

In our study, the results of correlation analysis of ASA score to SAS and APACHE II are statistically significant and the APACHE II score has a higher estimated mortality than the SAS and the P-POSSUM score.

As a measure of resource consumption, LOS is an essential consequence. Determining which factors contribute to an increased LOS could provide insight on how to cut expenses and improve care delivery. Preoperative, intraoperative, and postoperative variables are all linked to prolonged LOS [38,39]. Although preoperative characteristics were independently related with a prolonged LOS, the intraoperative process of care and postoperative adverse events were the factors that generated the greatest risk for a prolonged LOS [40]. Efforts should be made to improve the perioperative care process and to reduce postoperative complications. In patients with multiple comorbidities and those who require major surgery, those with high surgical and ASA scores may indicate a prolonged LOS. Balthazar et al. demonstrated a positive correlation between SAS and ASA class with LOS, suggesting that either score may be useful [41]. In our study, predicted LOS was correlated with surgical scores and ASA scale, validating the usefulness of scores in surgery and anesthesia. LOS can also estimate a calculation in terms of hospital expenses and the percentage of hospital beds occupied [38,42]. McDonald et al. published a study that supports the link between ASA class and LOS, concluding that ASA status is a powerful predictor of LOS in patients undergoing surgery fixation of ankle fractures [42]. The significant difference between the predicted and actual LOS in our study can be attributed to the need for safety given by strict supervision of the patient in a controlled environment. Prolonged LOS indicate the need for a more efficient patient classification system that can also predict potential surgical and anesthetic risks that could lengthen the patient’s hospital stay.

In our paper, the results of correlation analysis of ASA score to P-POSSUM, SAS, and APACHE II are statistically significant, but for improving the quality of patient care, preventing unwanted complications, and for a correct patient information about the risk of the surgery, we need a scoring system that is easy to use and can assess both anesthetic and surgical risk of the patient. On the other hand, to correctly inform the patient about the risk of the surgery and improve the quality of patient care, we need a scoring system that is easy to use and can assess both anesthetic and surgical risk.

Because the anesthetic-surgical act is complex, and interferes in terms of risks, complications, LOS and mortality, an ideal perioperative predictive score would have as many from the following: should not contain complicated items (such as expensive blood tests or values obtained through advanced monitoring), should be easy and quick to perform, have an easy scoring system, contain items regarding both surgery and anesthesia, and be valid in pre- intra and post-operative/anesthetic period.

Among the studied scores, the one that fulfilled the most the criteria for an ideal score was APACHE II, due to its physiological parameters that are partially superimposable to ASA score [43].

This pilot study has some limitations, such as the single-center nature of the study and the low number of included patients. Further research is needed.

## 5. Conclusions

Anesthetic, surgical, and severity scores, used together, provide clearer information about mortality, morbidity, and LOS.

ASA scale, associated with surgical scores and severity scores, presents a better image of the patient’s progress in the perioperative period. In our study, APACHE II is the best predictor of mortality, followed by P-POSSUM and SAS. P-POSSUM score and ASA scale may be complementary in terms of preoperative physiological factors, providing valuable information for postoperative outcomes. In order to obtain these results, a larger number of studied patients is required.

This draws attention to the need for a more comprehensive score, which should include both anesthetic and surgical risk and should also have the advantage of predicting morbidity, mortality, LOS, and the need for postoperative ICU admission.

## Figures and Tables

**Table 1:** Median values for length of stay (LOS) and predicted LOS—descriptive statistics.

	Minimum	Maximum	Percentiles			IQR
			25th	50th (Median)	75th	
LOS	0.20	30.00	5.00	9.00	11.00	6.00
Predicted LOS	0.00	30.00	2.00	4.00	6.00	4.00

**Table 2:** Estimated mortality of P-POSSUM, APACHE II, and SAS.

	Minimum	Maximum	Percentiles			IQR
			25th	50th (Median)	75th	
P-POSSUM mortality rate	0.20	22.70	0.40	1.35	4.87	4.48
APACHE II mortality rate	4.00	40.00	4.00	8.00	15.00	11.00
SAS mortality rate	0.00	14.00	1.00	1.00	1.02	0.02

**Table 3:** Correlation between predicted LOS and POSSUM, APACHE II, ASA scale.

Spearman’s Rho		POSSUM	APACHE II	ASA
Predicted LOS	Correlation Coefficient	0.433	0.454	0.676
	Sig. (Two-tailed)	0.002	0.001	0.000

**Table 4:** Negative correlation between predicted LOS, real LOS, ASA class, and SAS.

Spearman’s Rho		LOS	Predicted LOS	ASA
SAS	Correlation Coefficient	−0.326	−0.486	−0.446
	Sig. (2-tailed)	0.021	0.000	0.001

## Data Availability

The data used for this study can be found in the database of the Târgu Mureş County Emergency Clinical Hospital, Mureş Romania.
